# Association studies in outbred mice in a new era of full-genome sequencing

**DOI:** 10.1007/s00335-012-9409-z

**Published:** 2012-07-31

**Authors:** Binnaz Yalcin, Jonathan Flint

**Affiliations:** 1 Center for Integrative Genomics, University of Lausanne, Lausanne, Switzerland; 2 Institute of Genetics and Molecular and Cellular Biology, 67404 Illkirch, France; 3 Wellcome Trust Centre for Human Genetics, Roosevelt Drive, Oxford, OX3 7BN UK

## Abstract

Thousands of loci that contribute to quantitative traits in outbred crosses of mice have been reported over the last two decades. In this review we discuss how outbred mouse populations can be used to map and identify the genes and sequence variants that give rise to quantitative variation. We discuss heterogeneous stocks, the diversity outbred, and commercially available outbred populations of mice. All of these populations are descended from a small number of progenitor strains. The availability of the complete sequence of laboratory strains means that in many cases it will be possible to reconstruct the genomes of the outbred animals so that in a genetic association study we can detect the effect of all variants, a situation that has so far eluded studies in completely outbred populations. These resources constitute a major advance and make it possible to progress from a quantitative trait locus to a gene at an unprecedented speed.

## Introduction

In this review we discuss the use of outbred mice to map the genetic basis of complex traits and consider how recent advances in full-genome sequencing technologies have aided this endeavour. Most of our examples are drawn from heterogeneous stocks (HS) (Talbot et al. 1999; Valdar et al. 2006) and commercial outbreds (CO) (Yalcin et al. 2010), but there are new resources [such as the diversity outbred (DO) (Svenson et al. 2012)] that are likely to make an impact. We start though by considering in general terms why mapping in an outbred population is useful.

Many mouse geneticists, used to the availability of inbred strains and the ability they present to exclude genetic variation from their experiments, shrink from the mention of using an outbred. A 2005 review of the origins and uses of outbreds concluded that “we think that most papers that are currently published on the back of outbred stocks should be using inbred lines and any experimental plans, including grant applications and papers, based on outbred stocks need to be fully justified to avoid wasting animals and funding” (Chia et al. 2005). Nevertheless, it is also clear that in many ways inbred animals are unusual: they are less vigorous than outbreds and they lack the buffering effects of heterozygosity so that they suffer the consequences of recessive alleles.

The geneticist’s justification for the use of outbred stocks is that they solve a problem frequently met by those trying to map complex traits. Complex traits are those that arise from a combination of genetic and environmental effects. In this sense, most common diseases and quantitative traits of biomedical interest qualify as complex phenotypes. Attempts to identify the causative genetic variants often assume that starting from mapping a locus, using, for example, a cross between two inbred strains, variants can then be isolated on a homogeneous genetic background through the creation of congenics (Flint et al. 2005; Flint 2010). Variants are considered to act as Mendelian alleles of small effect, or low penetrance. While indeed there are examples where this approach has worked, they are far outnumbered by those where it has not worked. There are two reasons why this is so.

The first is that mapping using standard resources rarely gives gene-level resolution. This is particularly true of crosses between inbred strains where a locus that contributes to variation in a quantitative trait (a quantitative trait locus or QTL) is typically mapped to a region of about half a chromosome. Poor mapping resolution by itself need not vitiate the use of a congenic strategy. The problem is that poor resolution conflates the signal from a number of small-effect neighbouring loci into one locus; a number of independent small effects acting in the same direction are then misinterpreted as one large signal, and small effects acting in opposite directions cancel each other out, so that the locus is missed. The true situation emerges only with higher-resolution mapping so that congenic experiments, planned on the basis of the low-resolution mapping, are frustrated. There are a large number of examples of this, including QTLs influencing seizures (Legare et al. 2000), obesity (Stylianou et al. 2004), growth (Christians and Keightley 2004), blood pressure (Ariyarajah et al. 2004; Alemayehu et al. 2002; Garrett and Rapp 2002a, b; Frantz et al. 2001), diabetes (Podolin et al. 1998), antibody production (Puel et al. 1998), and infection (Bihl et al. 1999). One caveat that should be mentioned here is the possible presence of strain-specific hotspots (cluster of narrow regions) of recombination which may limit mapping resolution. The genomes of outbreds derived from a small set of progenitor strains may possess a particularly nonrandom distribution of hotspots. For instance, variation in *Prdm9* (*PR domain zinc finger protein 9*) is known to affect recombination activity in primates and mice (Baudat et al. 2010; Myers et al. 2010), and five alleles differing in the number of zinc finger repeats were found in 20 mouse strains (Parvanov et al. 2010). These differences may reflect differences in hotspot distribution (Brunschwig et al. 2012) in some regions of the genome.

The second reason is the frequent finding that the effect of a locus will depend on its genetic background. For example, in disease models a susceptibility locus may become a resistance locus and vice versa. Thus, when a locus found in a mapping experiment is placed on a different genetic background (e.g., during the construction of a congenic), its effect may increase, decrease, reverse in direction, or even disappear completely.

The use of outbreds can deal with both problems. First, they have accumulated numerous recombinations so that the correlation between genotypes [as measured by linkage disequilibrium (LD)] is typically much lower than that in many other mapping resources. This means that the correlation between the causative variant and markers decreases sharply with increasing distance thereby providing high-resolution mapping. Second, in an outbred the effects of genetic background are diminished. Because there are so many loci segregating, some of which increase and some of which decrease the phenotype, the joint effect will tend towards zero. By contrast, in a single genetic background, as happens when a locus is isolated on one strain, there is more chance that the effect of single or a few alleles will be manifest.

The mouse has three key features enabling investigators to map quantitative trait or disease-related genes. The first is the large number (hundreds) of well-characterized inbred strains of laboratory mice. The second is the large amount of phenotypic diversity among these strains, including behavioural and physiological differences. The Mouse Phenome Project (Bogue and Grubb 2004; Paigen and Eppig 2000) aims at collecting phenotypic data on inbred strains of mice (http://www.jax.org/phenome/) (Bogue et al. 2007; Grubb et al. 2009; Maddatu et al. 2012). To date, 1,804 phenotypic measurements have been collected in over 100 inbred mouse strains. The third key feature for mapping genes in the mouse is the development of complete catalogues of genomic variation maps between inbred strains of mice (Wade and Daly 2005) (reviewed in (Yalcin et al. 2012)). Several maps of single-nucleotide polymorphisms (SNPs) had been reported using array-based technologies, including a map with 0.55 million (M) SNPs [the Mouse Diversity array (Yang et al. 2009)] in 100 classical strains and 62 wild-derived laboratory strains (Yang et al. 2011), a map with 0.12 M SNPs in 94 inbred mouse strains (Kirby et al. 2010), and one with 8.3 M SNPs in 15 inbred strains (Frazer et al. 2007). Remarkably, the use of next-generation sequencing technologies allowed the identification of almost ten times more sequence variants in 17 genomes, including 56.7 M unique SNPs, 8.8 M short indels (<100 bp), and 0.28 M structural variants (Keane et al. 2011).

We describe below three outbred mouse resources for genetic mapping: heterogeneous stocks (HS) (Talbot et al. 1999; Valdar et al. 2006), the mouse diversity outbred (DO) population (Svenson et al. 2012), and commercial outbreds (CO) (Yalcin et al. 2010). All three exploit the accumulation of historical recombination to provide increased mapping resolution. Combined with full-genome sequences (Keane et al. 2011; Yalcin et al. 2011), these strategies allow progress from a QTL to a gene at an unprecedented speed (Box 1).

## Association studies using heterogeneous stocks (HS)

**Fig. 1:**
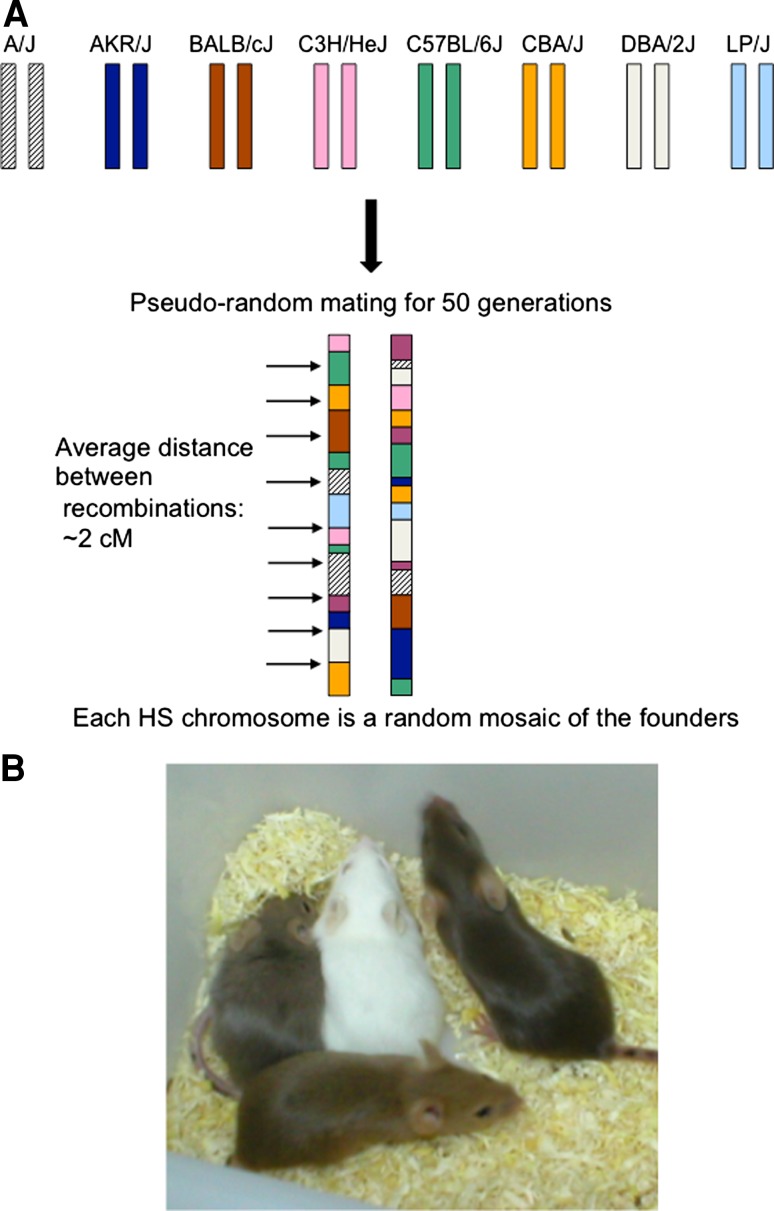
The Northport heterogeneous stock (HS). **a** Strategy to make the experimental cross. **b** Photo of four HS mice

Heterogeneous stocks are derived from inbred strains. After many generations of pseudorandom breeding, sufficient recombinants are introduced to enable high-resolution mapping. Chromosomes in HS animals are a random mosaic of the eight founders (Fig. 1a). Mice are unique and genetically heterogeneous, as illustrated in Fig. 1b with HS mice that have a different coat colour. Two HS have been used for mouse mapping experiments: the older of the two (Boulder HS) has been breeding for more than 60 generations and is derived from the following eight strains: A/J, AKR/J, BALB/cJ, C3H/HeJ, C57BL/6J, DBA/2J, I/LnJ, and RIII/DmMobJ (McClearn et al. 1970). The second heterogeneous stock (Northport HS) (Demarest et al. 2001) is derived from A/J, AKR/J, BALB/cJ, C3H/HeJ, C57BL/6J, CBA/J, DBA/2J, and LP/J strains.

Most alleles segregating in a HS are those present in the progenitors (there will be relatively few new mutations accumulating over time). This has a number of consequences. Perhaps, most importantly, it means that the derivation of sequence in a HS animal can be traced back to its progenitors so that the mosaic structure of each chromosome can be reconstructed using probabilistic ancestral haplotype reconstruction, with a sufficiently dense set of markers (Mott et al. 2000). Typically for a HS that has been through more than 50 generations of breeding, this requires approximately 10,000 markers, far fewer than is needed to capture common variation in a fully outbred population like our own. It also means that there will be far fewer variants with minor allele frequency (MAF) less than 5 % than in a fully outbred population. When a HS is first created, the rarest allele will have a frequency of 12.5 % (1/8), and although drift will drive some alleles lower, the breeding schedule is designed to maintain allele frequencies as high as possible. If the HS was maintained as a large randomly breeding population, the expected time to loss of an allele with an initial frequency of 12.5 % is about 450 generations, and under breeding regimes that maintain heterozygosity, the time to fixation will be even longer. Thus, genetic analysis of a HS is an analysis of the effects of common variants.

However, analysis of outbred populations of mice such as the HS is complicated, some might say to such a degree that it undermines its value for genetic mapping by the presence of population structure. Unlike a fully outbred population consisting of unrelated individuals, HS animals are related to each other to differing degrees, so that some could be as close as first cousins and others much more distantly removed. Simple association mapping will generate false positives if structure is ignored: long-range correlations between genetic markers occur. In fact, it is sometimes possible to predict the genotype of a marker on one chromosome by the genotype on another. These long-range correlations might be due to partial fixation of pairs of haplotype blocks within subsets of the population, or they might be an indication that the breeding history of the population was not optimal. There are ways to deal with population structure in a HS, e.g., using mixed-model association (Kang et al. 2008) or model-averaging approaches (Valdar et al. 2009), but to maximize power it is better to carry out association in the absence of population structure.

A large number of QTLs have now been mapped in HS populations (Talbot et al. 1999, 2003; Valdar et al. 2006; Hitzemann et al. 2002; Yalcin et al. 2004). In one study of over 100 phenotypes, 843 QTLs were identified in a population of over 2,000 mice (Valdar et al. 2006). The average mapping resolution is about 3 megabases (Mbp), providing approximately 30 candidate genes per locus. This is a good starting point for further studies, and in one instance it has led to the identification of a quantitative trait gene involved in behaviour: *Rgs2*, a regulator of G-protein signalling. *Rgs2* was shown by quantitative complementation to be involved in modulating anxious behaviour (Yalcin et al. 2004). However, HS do not deliver gene-level resolution.

## The diversity outbred (DO)

The diversity outbred (Svenson et al. 2012) [reviewed in (Churchill et al. 2012)] is a newly developed mouse population derived from progenitor lines of the Collaborative Cross (Chesler et al. 2008) [reviewed in (Welsh et al. 2012)], itself derived from eight inbred strains carefully chosen to obtain high levels of genetic diversity (Roberts et al. 2007). The DO is designed to provide high-resolution mapping. As with other outbred populations, each animal is genetically unique, but the DO has the advantage in that segregating allelic variants are present in the recombinant inbred strains of the Collaborative Cross, providing opportunities for replication and for additional experimental designs that are available to other resources. Opportunities afforded by the dual nature of CC and DO have yet to be fully explored.

Initial data from the DO show that it does have the expected properties of high-resolution mapping and high genetic diversity (Svenson et al. 2012). At generations 4 and 5 the distance between recombination events was 5.9 and 5.6 cM, respectively, and the number of informative recombination events per animal is expected to increase by about 28 per generation. Heterozygosity, assessed by a mouse SNP chip designed for experiments using CC mice (thus avoiding biases in SNP selection), was about 40 %. How does the DO perform in mapping studies? Using about 100 animals, it was possible to identify significant QTLs for 11 phenotypes, with confidence intervals under 3 Mbp (Svenson et al. 2012). Later generations should deliver much higher resolution.

The DO is currently uniformly structured and is maintained from 175 breeding pairs (in comparison, HS are maintained with about 40 breeding pairs). Each subsequent generation is created by selecting two offspring, one female and one male, at random. However, as in the HS, DO animals will be related to each other by different degrees so that, again, genetic mapping needs to take into account genetic relatedness.

## Commercial outbreds (CO)

Mouse vendors such as Harlan Sprague Dawley (Hsd), Charles River Laboratories (Crl), and Taconic Farms (Tac) maintain large colonies of outbred crosses of mice consisting of up to 10,000 animals per colony that have accumulated many recombination events. These commercially available outbred crosses are used primarily by pharmaceutical companies and until recently were not considered as reference genetic populations mainly because of their poor genetic characterization (Chia et al. 2005; Festing 1999).

In recent years, there has been an increasing awareness of the utility of CO mice as resources for fine-scale genetic association studies. Table 1 lists studies that used CO mice, with relevant information about colony, locus, and phenotype. Early examples include the CD1 mice at Charles River Laboratories in Italy [Crl:CD1(ICR)-IT as defined by our outbred strain nomenclature (Yalcin et al. 2010)] that were used to analyse the susceptibility allele at the pulmonary adenoma susceptibility 1 (*Pas1*) locus in *Kras2* and *Pthlh* genes (Manenti et al. 2003). Several years ago we used the MF1 colony from Harlan Sprague Dawley (HsdOla:MF1-UK) to fine map a QTL influencing anxiety (Yalcin et al. 2004) (note that in 2005 this colony underwent a major population bottleneck caused by a bacterial infection of *Helicobacter hepaticus*). Other examples include a genome-wide association analysis of hepatic gene expression traits in the HsdOla:MF1-US colony (Ghazalpour et al. 2008), a genetic analysis of colonies of Crl:CD1(ICR)-US at several locations (Aldinger et al. 2009), and a fine mapping of loci influencing cardiovascular disease risk using the BomTac:NMRI-DK colony (Zhang et al. 2012).

**Table 1:** Genetic studies using commercial outbred (CO) populations of mice

Colony	Locus/trait	Reference
Crl:CD1(ICR)-IT	Pulmonary adenoma susceptibility 1	Manenti et al. (2003)
HsdOla:MF1-UK	Anxiety susceptibility locus	Yalcin et al. (2004)
HsdOla:MF1-US	Hepatic gene expression traits	Ghazalpour et al. (2008)
Crl:CD1(ICR)-US	Genetic evaluation of colonies	Aldinger et al. (2009)
BomTac:NMRI-DK	Cardiovascular disease risk	Zhang et al. (2012)
Crl:CFW(SW)-US_P08	Haematological traits	Yalcin et al. (2010)
HsdWin:CFW-NL	Haematological traits	Yalcin et al. (2010)
HsdWin:NMRI-NL	Haematological traits	Yalcin et al. (2010)

To our surprise, we found that there were dozens of CO colonies available, not just a handful as we initially thought. Therefore, we set out to explore the usefulness for association studies of all crosses available at the time of our study (2007–2009) (Yalcin et al. 2010). We surveyed genetic variation in 66 CO colonies. This study came to two important conclusions. The first is that some CO populations of mice have haplotype blocks of less than 100 kbp, enabling gene-level mapping resolution and making these populations ideal for association studies. Other CO populations were genetically less diverse and had large LD blocks; rare cases had almost completely inbred genomes. As proof of principle, we used three colonies [Crl:CFW(SW)-US_P08, HsdWin:CFW-NL, and HsdWin:NMRI-NL] of the 66 evaluated and refined several previously identified QTLs (Yalcin et al. 2010) (Table 1). Notably, we identified the molecular variation that influences the ratio of CD4^+^/CD8^+^ in T lymphocytes.

The second important finding is that almost all of the genetic variants in the CO populations can be found in classical laboratory strains. We were able to show this from haplotype reconstruction and confirmed it in one population by genome-wide sequencing support. This means that variants in the CO mice can be modelled as descending from inbred progenitors, and it becomes possible to impute the sequence of any CO mouse from the sequences of inbred strains. The full catalogue of sequence variation can thus be obtained by sequencing the inbred strains presumed to be founders for it and genotyping the stock at a skeleton of SNPs. This means that in a genetic association study we can detect the effect of all variants, a situation that has so far eluded studies in completely outbred populations.

Because of these two important findings, i.e., gene-level mapping resolution and known ancestry, we have proposed CO stocks as a novel powerful resource for fine-resolution genetic mapping. Not all of the CO stocks are suitable for association studies, with a few that should clearly be avoided. However, it is important to make a good choice of the population to use. Here we will highlight two genetic measurements that we used to classify these stocks: LD decay radius and mean MAF (Yalcin et al. 2010). Basically, the lower the LD decay radius, the higher the mapping resolution, and the lower the mean MAF, the higher the number of animals to be used. For example, our evaluation of BomTac:NMRI-DK yielded a LD decay radius of 1.07 Mbp and a mean MAF of 0.068. If mapping resolution is important in a genetic experiment, BomTac:NMRI-DK might not be the best population from which to choice, as used in a recent study (Zhang et al. 2012). Because MAF is small, a large number (in the thousands) of animals should be used to map small genetic effects.

CO populations contain a relatively small subset of all known variations in the mouse, a finding that has both advantages and disadvantages for genetic studies. Since the populations are subsets of the same set of alleles, mapping for a trait of interest might work in one population but might not work in another. We found that the extent of genetic variation between colonies is remarkably large. Fst, a measure of variation within and between populations [reviewed in (Holsinger and Weir 2009)], is 0.45 [in contrast, human population values are typically <0.05 (Reich et al. 2009)]. However, the relatively limited genetic diversity of the outbred colonies also means that they do not model a fully outbred population; nor can they be used to assess the effect of all variants present in mouse populations.

Table 2 provides the current list of CO strains of mice. It should be noted that there have been colony movements and changes since our last study (Yalcin et al. 2010). Over time, vendors can stop providing a specific colony or expand a colony at a different geographical location. For example, in 2009, Crl:OF1 used to be maintained at two locations (Hungary and France), but it is now available only from France. Other examples include HsdWin:NMRI and Hsd:NIHSBC, for which breeding stopped at the German and Israeli Harlan sites, respectively. In other cases such as Hsd:ICR(CD-1), a new colony has been started in Korea. In one case, HsdWin:CFW, cryopreservation has been chosen as a way to keep the line. Future studies using HsdWin:CFW will have to re-evaluate the amount of genetic diversity and the potential population bottleneck that occurred during cryopreservation.

**Table 2:** Current list of commercial outbred (CO) populations of mice

Vendor	Strain	Country
B & K Universal	BK:W	UK
Charles River	Crl:CD1(ICR)	US, IT, FR, DE
	Crl:CF1	US
	Crl:CFW(SW)	US
	Crl:MF1	UK
	Crl:NMRI(Han)	FR, DE
	Crl:OF1	FR
	Crlj:CD1(ICR)	JP
Elevage Janvier	RjHan:NMRI	FR
	RjOrl:Swiss	FR
Harlan	ClrHli:CD1	IL
	Hsd:ICR(CD-1)	US, UK, NL, IL, IT, MX, KP
	Hsd:ND4	US
	Hsd:NIHS	US, UK
	Hsd:NSA(CF1)	US
	HsdHu:SABRA	IL
	HsdIco:OF1	IT
	HsdOla:MF1	UK
	HsdOla:TO	UK
	HsdWin:NMRI	UK, NL
Hilltop	Hla:(ICR)CVF	US
NOVA-SCB	Sca:NMRI	DE
SAGE	Aai:ICR	US
	Aai:SW	US
Simonsen	Sim:(SW)fBR	US
Taconic	BomTac:NMRI	DK
	IcrTac:ICR	US
	NTac:NIHBS	US
	Tac:SW	US

There are currently nine vendors including *BK* B & K Universal, *Crl* Charles River Laboratories, *Rj* Elevage Janvier, *Hsd* Harlan Sprague Dawley, *Hla* Hilltop Lab Animals, NOVA-SCB [previously known as *Sca* Scanbur], *SAGE* Sigma Advanced Genetic Engineering, *Sim* Simonsen, and *Tac* Taconic Farms. Note that Ace Animals was bought in 2011 by SAGE, and the Research and Consulting Company (known as RCC) closed in 2010

**Box 1:** Online resources for genetic studies using outbred crosses of mice

Resource	Web URL
Northport heterogeneous stock (HS)	http://gscan.well.ox.ac.uk
Commercially available outbreds (CO)	http://www.well.ox.ac.uk/flint-old/outbreds.shtml
QTL mapping using HS mice	http://gscan.well.ox.ac.uk/gsBleadingEdge/wwwqtl.cgi
QTL mapping using CO mice	http://www.well.ox.ac.uk/flint-old/outbreds/QTL.MAPPING
Full-genome sequencing of CO mice	http://mus.well.ox.ac.uk/mus/outbred

## The availability of full-genome sequences

**Fig. 2:**
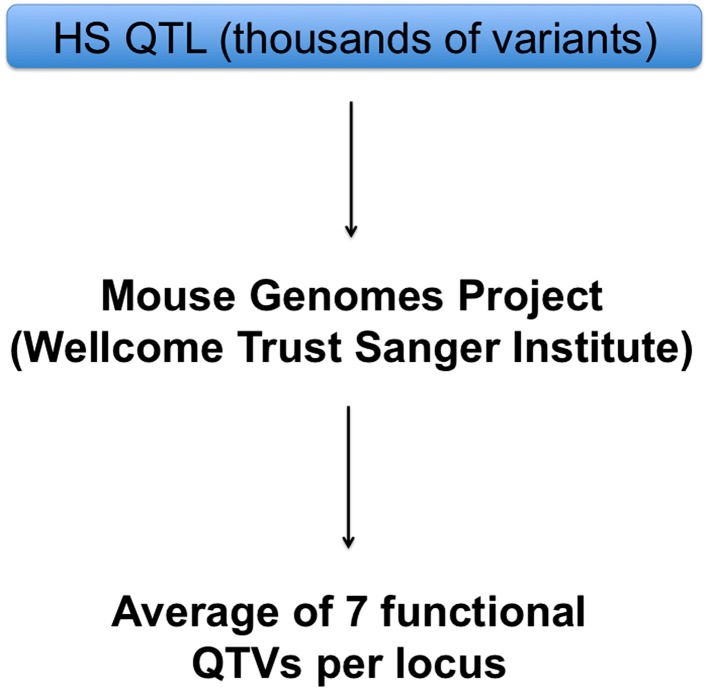
Strategy for the rapid identification of quantitative trait variants (QTVs) in HS mice

The Mouse Genomes Project (Keane et al. 2011) has made a major difference to the identification of the causative quantitative trait genes: when populations are descended from known progenitors, there is no need to sequence genes or other genomic regions. There are already cases where the availability of the sequence data has led to advances. For example, at each of the 843 QTLs previously identified in the HS (Valdar et al. 2006), we were able to apply a test that discriminates between variants that could be functional and those that are not. The test is explained in detail in (Yalcin et al. 2005) and is based primarily on the strain distribution pattern of the variant. This analysis allowed us to identify on average seven functional variants across 718 of the 843 QTLs (Keane et al. 2011) (Fig. 2). In addition, at 10 % of the QTLs there was one single functional variant detected and therefore could unambiguously be assigned as a causative variant (Keane et al. 2011).

Similarly, in the DO the sequence helped refine the location and identify functional variants. While mapping a QTL influencing total plasma cholesterol, founder haplotypes were identified from strains 129S1/SvImJ, WSB/EiJ, and NZO/HILtJ within a 2-Mbp region. This region included 11 protein-coding genes and 32,196 SNPs. However, only seven were consistent with the allele effect pattern, a remarkable reduction in the number of potentially causal variants (Svenson et al. 2012).

It is a very exciting time for genome-wide association studies in mice. Major advances in sequencing technologies have allowed for the first time the identification of quantitative trait genes and quantitative trait variants at an unprecedented speed, and have made possible the investigation of the genetic architecture of quantitative traits (Keane et al. 2011). The use of CO populations of mice in conjunction with complete genome sequence data of the predicted founder strains can reduce the list of candidate variants at each QTL to small numbers of causal variants. In addition to the gene-level mapping resolution, CO populations of mice also offer a unique opportunity to explore the “missing heritability” problem that has been reported in human association studies (Parker and Palmer 2011). Because their effective population size is large, rare alleles have been accumulated in these populations and could be used as paradigms for examining how these rare variants impact on phenotypic variation.

## References

[CR1] Aldinger KA, Sokoloff G, Rosenberg DM, Palmer AA, Millen KJ (2009). Genetic variation and population substructure in outbred CD-1 mice: implications for genome-wide association studies. PLoS One.

[CR2] Alemayehu A, Breen L, Krenova D, Printz MP (2002). Reciprocal rat chromosome 2 congenic strains reveal contrasting blood pressure and heart rate QTL. Physiol Genomics.

[CR3] Ariyarajah A, Palijan A, Dutil J, Prithiviraj K, Deng Y, Deng AY (2004). Dissecting quantitative trait loci into opposite blood pressure effects on Dahl rat chromosome 8 by congenic strains. J Hypertens.

[CR4] Baudat F, Buard J, Grey C, Fledel-Alon A, Ober C, Przeworski M, Coop G, de Massy B (2010). PRDM9 is a major determinant of meiotic recombination hotspots in humans and mice. Science.

[CR5] Bihl F, Brahic M, Bureau JF (1999). Two loci, *Tmevp2* and *Tmevp3*, located on the telomeric region of chromosome 10, control the persistence of Theiler’s virus in the central nervous system of mice. Genetics.

[CR6] Bogue MA, Grubb SC (2004). The mouse phenome project. Genetica.

[CR7] Bogue MA, Grubb SC, Maddatu TP, Bult CJ (2007). Mouse phenome database (MPD). Nucleic Acids Res.

[CR8] Brunschwig H, Levi L, Ben-David E, Williams RW, Yakir B, Shifman S (2012). Fine-scale map of recombination rates and hotspots in the mouse genome. Genetics.

[CR9] Chesler EJ, Miller DR, Branstetter LR, Galloway LD, Jackson BL, Philip VM, Voy BH, Culiat CT, Threadgill DW, Williams RW (2008). The Collaborative Cross at Oak Ridge National Laboratory: developing a powerful resource for systems genetics. Mamm Genome.

[CR10] Chia R, Achilli F, Festing MF, Fisher EM (2005). The origins and uses of mouse outbred stocks. Nat Genet.

[CR11] Christians JK, Keightley PD (2004). Fine mapping of a murine growth locus to a 1.4-cM region and resolution of linked QTL. Mamm Genome.

[CR12] Churchill GA, Munger SC, Swenson KL (2012) The diversity outbred mouse population. Mamm Genome. doi:10.1007/s00335-012-9414-2 10.1007/s00335-012-9414-2 PMC3524832 22892839

[CR13] Demarest K, Koyner J, McCaughran J, Cipp L, Hitzemann R (2001). Further characterization and high-resolution mapping of quantitative trait loci for ethanol-induced locomotor activity. Behav Genet.

[CR14] Festing MF (1999) Warning: the use of heterogeneous mice may seriously damage your research. Neurobiol Aging 20:237–244; discussion 245–236 10.1016/s0197-4580(99)00040-8 10537033

[CR15] Flint J (2010). Mapping quantitative traits and strategies to find quantitative trait genes. Methods.

[CR16] Flint J, Valdar W, Shifman S, Mott R (2005). Strategies for mapping and cloning quantitative trait genes in rodents. Nat Rev Genet.

[CR17] Frantz S, Clemitson JR, Bihoreau MT, Gauguier D, Samani NJ (2001). Genetic dissection of region around the Sa gene on rat chromosome 1: evidence for multiple loci affecting blood pressure. Hypertension.

[CR18] Frazer KA, Eskin E, Kang HM, Bogue MA, Hinds DA, Beilharz EJ, Gupta RV, Montgomery J, Morenzoni MM, Nilsen GB (2007). A sequence-based variation map of 8.27 million SNPs in inbred mouse strains. Nature.

[CR19] Garrett MR, Rapp JP (2002). Two closely linked interactive blood pressure QTL on rat chromosome 5 defined using congenic Dahl rats. Physiol Genomics.

[CR20] Garrett MR, Rapp JP (2002). Multiple blood pressure QTL on rat chromosome 2 defined by congenic Dahl rats. Mamm Genome.

[CR21] Ghazalpour A, Doss S, Kang H, Farber C, Wen PZ, Brozell A, Castellanos R, Eskin E, Smith DJ, Drake TA, Lusis AJ (2008). High-resolution mapping of gene expression using association in an outbred mouse stock. PLoS Genet.

[CR22] Grubb SC, Maddatu TP, Bult CJ, Bogue MA (2009). Mouse phenome database. Nucleic Acids Res.

[CR23] Hitzemann R, Malmanger B, Cooper S, Coulombe S, Reed C, Demarest K, Koyner J, Cipp L, Flint J, Talbot C (2002). Multiple cross mapping (MCM) markedly improves the localization of a QTL for ethanol-induced activation. Genes Brain Behav.

[CR24] Holsinger KE, Weir BS (2009). Genetics in geographically structured populations: defining, estimating and interpreting F(ST). Nat Rev Genet.

[CR25] Kang HM, Zaitlen NA, Wade CM, Kirby A, Heckerman D, Daly MJ, Eskin E (2008). Efficient control of population structure in model organism association mapping. Genetics.

[CR26] Keane TM, Goodstadt L, Danecek P, White MA, Wong K, Yalcin B, Heger A, Agam A, Slater G, Goodson M (2011). Mouse genomic variation and its effect on phenotypes and gene regulation. Nature.

[CR27] Kirby A, Kang HM, Wade CM, Cotsapas C, Kostem E, Han B, Furlotte N, Kang EY, Rivas M, Bogue MA (2010). Fine mapping in 94 inbred mouse strains using a high-density haplotype resource. Genetics.

[CR28] Legare ME, Bartlett FS, Frankel WN (2000). A major effect QTL determined by multiple genes in epileptic EL mice. Genome Res.

[CR29] Maddatu TP, Grubb SC, Bult CJ, Bogue MA (2012). Mouse phenome database (MPD). Nucleic Acids Res.

[CR30] Manenti G, Galbiati F, Noci S, Dragani TA (2003). Outbred CD-1 mice carry the susceptibility allele at the pulmonary adenoma susceptibility 1 (*Pas1*) locus. Carcinogenesis.

[CR31] McClearn GE, Wilson JR, Meredith W, Lindzey G, Thiessen D (1970). The use of isogenic and heterogenic mouse stocks in behavioral research. Contributions to behavior-genetic analysis: the mouse as a prototype.

[CR32] Mott R, Talbot CJ, Turri MG, Collins AC, Flint J (2000). A method for fine mapping quantitative trait loci in outbred animal stocks. Proc Natl Acad Sci USA.

[CR33] Myers S, Bowden R, Tumian A, Bontrop RE, Freeman C, MacFie TS, McVean G, Donnelly P (2010). Drive against hotspot motifs in primates implicates the *PRDM9* gene in meiotic recombination. Science.

[CR34] Paigen K, Eppig JT (2000). A mouse phenome project. Mamm Genome.

[CR35] Parker CC, Palmer AA (2011). Dark matter: are mice the solution to missing heritability?. Front Genet.

[CR36] Parvanov ED, Petkov PM, Paigen K (2010). *Prdm9* controls activation of mammalian recombination hotspots. Science.

[CR37] Podolin PL, Denny P, Armitage N, Lord CJ, Hill NJ, Levy ER, Peterson LB, Todd JA, Wicker LS, Lyons PA (1998). Localization of two insulin-dependent diabetes (*Idd*) genes to the *Idd10* region on mouse chromosome 3. Mamm Genome.

[CR38] Puel A, Mevel JC, Bouthillier Y, Decreusefond C, Fridman WH, Feingold N, Mouton D (1998). Identification of two quantitative trait loci involved in antibody production on mouse chromosome 8. Immunogenetics.

[CR39] Reich D, Thangaraj K, Patterson N, Price AL, Singh L (2009). Reconstructing Indian population history. Nature.

[CR40] Roberts A, Pardo-Manuel de Villena F, Wang W, McMillan L, Threadgill DW (2007). The polymorphism architecture of mouse genetic resources elucidated using genome-wide resequencing data: implications for QTL discovery and systems genetics. Mamm Genome.

[CR41] Stylianou IM, Christians JK, Keightley PD, Bunger L, Clinton M, Bulfield G, Horvat S (2004). Genetic complexity of an obesity QTL (*Fob3*) revealed by detailed genetic mapping. Mamm Genome.

[CR42] Svenson KL, Gatti DM, Valdar W, Welsh CE, Cheng R, Chesler EJ, Palmer AA, McMillan L, Churchill GA (2012). High-resolution genetic mapping using the mouse diversity outbred population. Genetics.

[CR43] Talbot CJ, Nicod A, Cherny SS, Fulker DW, Collins AC, Flint J (1999). High-resolution mapping of quantitative trait loci in outbred mice. Nat Genet.

[CR44] Talbot CJ, Radcliffe RA, Fullerton J, Hitzemann R, Wehner JM, Flint J (2003). Fine scale mapping of a genetic locus for conditioned fear. Mamm Genome.

[CR45] Valdar W, Solberg LC, Gauguier D, Burnett S, Klenerman P, Cookson WO, Taylor MS, Rawlins JN, Mott R, Flint J (2006). Genome-wide genetic association of complex traits in heterogeneous stock mice. Nat Genet.

[CR46] Valdar W, Holmes CC, Mott R, Flint J (2009). Mapping in structured populations by resample model averaging. Genetics.

[CR47] Wade CM, Daly MJ (2005). Genetic variation in laboratory mice. Nat Genet.

[CR48] Welsh CE, Miller DR, Manly KF, Wang J, McMillan L, Threadgill DW, Pardo-Manuel de Villena F (2012) Status and access to the Collaborative Cross Population. Mamm Genome. doi:10.1007/s00335-012-9410-6 10.1007/s00335-012-9410-6 PMC3463789 22847377

[CR49] Yalcin B, Willis-Owen SA, Fullerton J, Meesaq A, Deacon RM, Rawlins JN, Copley RR, Morris AP, Flint J, Mott R (2004). Genetic dissection of a behavioral quantitative trait locus shows that *Rgs2* modulates anxiety in mice. Nat Genet.

[CR50] Yalcin B, Flint J, Mott R (2005). Using progenitor strain information to identify quantitative trait nucleotides in outbred mice. Genetics.

[CR51] Yalcin B, Nicod J, Bhomra A, Davidson S, Cleak J, Farinelli L, Osteras M, Whitley A, Yuan W, Gan X et al (2010) Commercially available outbred mice for genome-wide association studies. PLoS Genet 6(9). pii: e1001085 10.1371/journal.pgen.1001085 PMC2932682 20838427

[CR52] Yalcin B, Wong K, Agam A, Goodson M, Keane TM, Gan X, Nellaker C, Goodstadt L, Nicod J, Bhomra A (2011). Sequence-based characterization of structural variation in the mouse genome. Nature.

[CR53] Yalcin B, Adams DJ, Flint J, Keane T (2012) Next generation sequencing of experimental mouse strains. Mamm Genome. doi:10.1007/s00335-012-9402-6 10.1007/s00335-012-9402-6 PMC3463794 22772437

[CR54] Yang H, Ding Y, Hutchins LN, Szatkiewicz J, Bell TA, Paigen BJ, Graber JH, de Villena FP, Churchill GA (2009). A customized and versatile high-density genotyping array for the mouse. Nat Methods.

[CR55] Yang H, Wang JR, Didion JP, Buus RJ, Bell TA, Welsh CE, Bonhomme F, Yu AH, Nachman MW, Pialek J (2011). Subspecific origin and haplotype diversity in the laboratory mouse. Nat Genet.

[CR56] Zhang W, Korstanje R, Thaisz J, Staedtler F, Harttman N, Xu L, Feng M, Yanas L, Yang H, Valdar W (2012). Genome-wide association mapping of quantitative traits in outbred mice..

